# Handling uncertainties inherited in life cycle inventory and life cycle impact assessment method for improved life cycle assessment of wastewater sludge treatment

**DOI:** 10.1016/j.heliyon.2019.e02793

**Published:** 2019-11-14

**Authors:** Isam Alyaseri, Jianpeng Zhou

**Affiliations:** a Department of Civil Engineering, Al-Muthanna University, 72001, Iraq; b Department of Civil Engineering, Southern Illinois University Edwardsville, IL, 62026-1800, USA

**Keywords:** Engineering, Civil engineering, Environmental science, Environmental assessment, Environmental engineering, Environmental impact assessment, Uncertainty, Wastewater sludge treatment, LCA, Life cycle impact assessment method, Data inventory

## Abstract

Life cycle assessment (LCA) has been used to evaluate environmental impacts of products or processes including wastewater treatment. Uncertainty has not received adequate attention in LCA studies. Uncertainty inherited in LCA steps such as the life cycle inventory (LCI) or the life cycle impact assessment (LCIA) method use is unavoidable, but it affects LCA outcomes and associated decision-making. The objective of this paper was to show the impact of uncertainty from LCI and LCIA method on LCA outcomes by using a case study base approach on wastewater sludge treatment processes. A qualitative analysis included setting criteria about what data to be included in LCI, characterization of data, differentiating between major and minor contributors in LCI modeling, evaluation of data quality indicators, setting achievable alternative scenarios, and selecting proper LCIA method were used, in addition to quantitative analysis included assigning most appropriate values for data gaps and proper distribution, and conducting probabilistic analysis to evaluate overall uncertainty. This research used a full-scale wastewater treatment plant in Missouri, USA for case study in which multiple hearth incineration (MHI) is the existing process, while fluid bed incineration (FBI) and anaerobic digestion (AD) were proposed as the alternatives. Using ReCipe method, the study revealed that variation in LCA results of MHI is 63.4% for a single end-point score of 57.9 mPt. On the two alternative processes, it is 54.6% probable that FBI would have more environmental impact than AD. The case study showed that the proposed steps were able to address issues of data uncertainty. Due to differences in characterization, normalization, and weighting factors, different LCIA methods may point out different conclusions and need to be addressed in evaluation.

## 1 Introduction

Life cycle assessment (LCA) has been used to evaluate environmental impacts of products or processes. LCA can assess the potential environmental impacts from all stages of a product or a process life cycle. In the wastewater treatment plants (WWTPs), LCA had been used to select the processes that have better environmental performance. LCA studies of wastewater treatment had been conducted to compare different stages of a treatment process, combinations of various treatment units, and varieties of treatment scenarios. A number of LCA studies analyzed practical application as case studies (e.g. Munoz et al., 2006; Hoibye et al., 2008; Pasqualino et al., 2009; and Alyaseri and Zhou, 2017). Many processes and new technologies were tested or evaluated using LCA as well (e.g. Ortiz et al., 2007; Munoz et al., 2007; Machado et al., 2007; and Park et al., 2008).

Although many efforts were made to incorporate uncertainty analysis in LCA studies, it is still uncommon practice (Lioyd and Ries, 2007). The impact of uncertainty on outcomes of LCA were not considered in many studies, including a number of comparative evaluations of different wastewater sludge management and treatment processes (such as Lederer and Rechberger, 2010; Hong et al., 2009; Pasqualino et al., 2009; Akwo, 2008; Tarantini et al., 2007; Svanstrom et al., 2005; and Suh and Rousseaux, 2002). Neglecting the impact of uncertainty in LCA studies may results in reducing confidence on LCA outcomes. Niero et al. (2014) showed that it is difficult to draw a robust conclusion between some wastewater treatment techniques if uncertainty analysis was not adequately addressed.

Many classifications for uncertainty can be found in literature (Heijungs and Huijbregts, 2004). Lioyd and Ries (2007) classified uncertainty in LCA into three classes: parameter, scenario, and model uncertainty. The parameter uncertainty concerns about data representativeness and may include uncertainty in the data relating to process inputs and environmental discharges, uncertainty in the data due to spatial and temporal variations, uncertainty due to technology characteristics, random errors, statistical variation, and uncertainty due to approximation. The scenario uncertainty included uncertainty in choices’ selection, valuation and weighting factors, time horizons, geographical scales, natural contexts, allocation procedures, waste handling scenarios, environmental thresholds, and expected technology trends. The model uncertainty included uncertainty in models for deriving emissions and characterization factors. Other types of uncertainty may include subjective judgment or volitional uncertainty, disagreement among stakeholders, systematic errors, and ambiguity of information (Heijungs and Huijbregts, 2004).

Quantifying uncertainties is essential and important for LCA studies (Van Zelm and Huijbregts, 2013). Finnveden et al. (2009) recommended using three approaches to address the issue of uncertainty: 1) a technical approach that develops data and models of improved quality through expanded researches; 2) a societal approach that builds consensus among stakeholders to develop the needed LCA data; and 3) a statistical approach that quantifies uncertainties and incorporates the impact of uncertainty in LCA outcomes and related decision-making process.

One commonly used method to reduce the impact of uncertainty on LCA outcomes is to decrease the boundaries of the system to be analyzed. In wastewater treatment processes, this can be done by limiting the LCA to a case study to assure the use of representative data as possible. This may be referred to as “case study base approach”. But even when dealing with one plant, the data collected will have variability. Also, more data still need to be collected to compensate missing data and build alternative scenarios which usually associated with the problem of variability in conditions. In the processes of wastewater treatment, the expected sources for variability may include: variability in the data collected due to spatial or temporal conditions, variability comes from inherent variations in the real world, variability in sources and variability in treatment conditions. Such data may also be outdated or miscalculated due to human errors. In addition, data uncertainty may also come from inaccurate measurement or approximation due to lack of data.

To handle LCA related uncertainties, Maurice et al. (2000) discussed three major methods: sensitivity analysis, qualitative assessment, and quantitative assessment. The sensitivity analysis is to identify major contributors in the LCA model, and evaluate how changes in the values of these major contributors affect the LCA outcomes. The qualitative assessment includes the characterization of data and sorting them according to the factors that may cause variabilities in LCA outcomes. The quantitative assessment is to apply appropriate statistical analysis that involve in intervals, scenario modeling, fuzzy data, analytical uncertainty, propagation, probabilistic simulation, and Bayesian statistics (e.g. Lioyd and Ries, 2007; Groen et al., 2014).

An LCA study typically consists of four stages: setting the scope and boundary of the study, developing life cycle inventory (LCI), conducting impact assessment, and interpreting results. Each LCA stage involves in various levels of uncertainty (ISO, 2006), which unavoidably affects LCA outcomes and the LCA-based decision-making. Uncertainties inherited in LCI data and the difference caused by choosing different life cycle impact assessment (LCIA) method can cause most significant impacts on outcomes of an LCA study (Lioyd and Ries, 2007; Schulze et al., 2001; and Leitão, 2016). Many sources such as outdated, incomplete, or incorrect data can contribute to uncertainty in LCI (Hendrickson et al., 2006). Hung and Ma (2009) showed that the uncertainty can vary between the LCIA methods used for assessment. Johnson et al. (2011) showed that the framework needed for incorporating uncertainty analysis in an LCA study may vary with LCA type. The objective of this research was to evaluate the impact of uncertainties associated with LCI and LCIA on LCA outcomes through case study of a full-scale wastewater sludge treatment processes, and provide a structured and systematic method for handling LCA uncertainties.

## 2 Methodology

### 2.1 Scope of the study

**Fig. 1:**
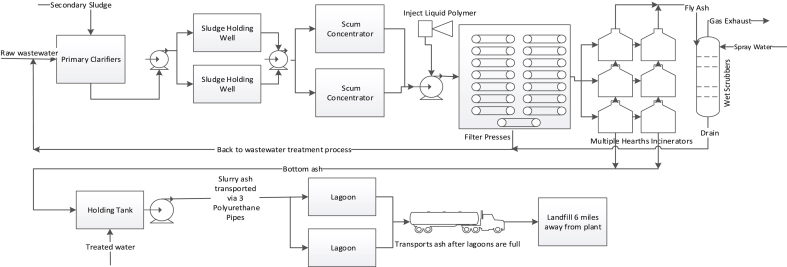
MHI wastewater sludge treatment process of Saint Louis BPWWTP (current process).

The wastewater sludge treatment process of the Bissell Point Wastewater Treatment Plant (BPWWTP) in Saint Louis, Missouri, USA was chosen for case study. As shown in Fig. 1, the BPWWTP sludge treatment process mainly consists of mechanical sludge dewatering by belt filter presses, multiple hearth incineration (MHI), bottom ashes to lagoons for drying then to landfill for final disposal. The LCI data that included both operation (e.g. wastewater flow rates) and facility installation (e.g. building materials) were collected directly from the BPWWTP. Commonly accepted data published in authoritative technical literatures were used to fill data gaps.

**Fig. 2:**
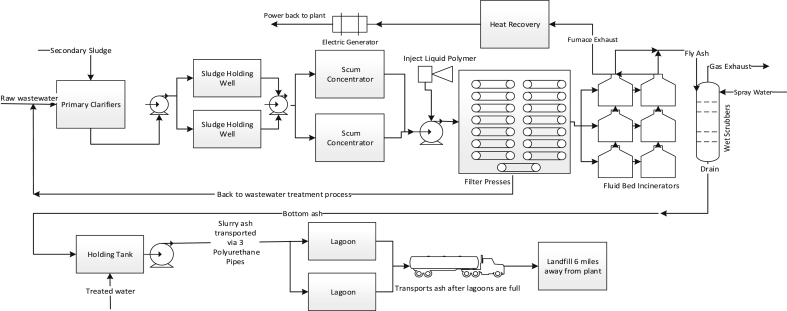
Wastewater sludge treatment process with fluid bed incineration (A proposed alternative).

**Fig. 3:**
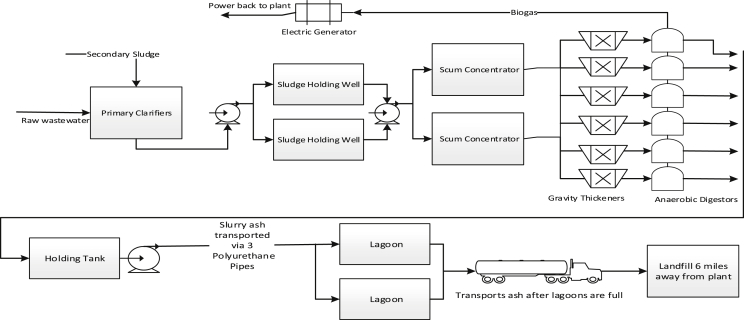
Wastewater sludge treatment process with anaerobic digestion (A proposed alternative).

For comparative analysis, two alternative treatment processes were developed for this research. The first alternative process, as shown in Fig. 2, mainly consists of sludge dewatering by belt filter presses, fluid bed incineration (FBI), bottom ashes to lagoons for drying then to landfill for final disposal. To limit variations, the same scrubber system currently in place will be used and no increase for air permit was assumed. The second alternative process, as shown in Fig. 3, mainly consists of gravity thickening of the sludge, anaerobic digestion (AD), digested sludge to lagoons for drying then to landfill for final disposal. The sludge that feed into these two alternative processes is the same as that to the current MHI process. For those operations and facility installation aspects of these two alternative processes that are identical to those of the MHI process (e.g. mechanical dewatering equipment, lagoons, landfills), that same data from BPWWTP were used. Where there were data gaps (e.g. data of FBI facility, gravity thickeners, AD tank, biogas production), commonly accepted data published in authoritative technical literatures were used. Examples (e.g. energy production from the AD process) describing how LCI data for proposed alternative processes were developed are discussed in the file “Supplementary Materials.doc”.

### 2.2 LCA functional unit

In LCA studies on sludge management, it is preferred to select a dry mass unit (e.g. one dry kg of untreated sludge) as a functional unit so every LCI can be normalized to it. The fraction of volatile solids (VS) in the sludge affects the operations and released emissions for the sludge treatment process. USEPA (1979) estimated the VS fraction of sludge from typical municipal WWTPs such as BPWWTP is in the range of 60–80%. Suh and Rousseaux (2002) and Tarantini et al. (2007) used 72% and 70% for VS, respectively. This research set 70% as the VS fraction of the studied sludge.

### 2.3 LCA boundary

LCA boundary defines the scope of LCI. The LCA study included materials for building various facilities of the treatment processes; energy, chemicals, and major supplies that are consumed or recovered during operation. For example, consumed natural gas and electricity, collected biogas by the AD process, used polymer for dewatering, fuels and equipment that are used to transport final residuals to landfill, landfill operations, and the land occupied by the treatment plant were included. However, the energy and fuels that were consumed for constructing the plant were excluded on basis on the studies by Hospido et al. (2005); Suh and Rousseaux (2002); Hong et al. (2009); and Murray et al. (2008) that these consumptions are relatively small amount when comparing to other LCI values. The office building, general maintenance, labor, dust and noises control, and auxiliary water usage were excluded from this study because these units are used in all of studied processes. Tarantini et al. (2007) recommended that, when two or more processes are compared, LCI doesn't need to include every unit that are identical and are used by all processes.

### 2.4 Characterization of LCI data

Hung and Ma (2009) showed that the primary uncertainty comes from LCI in a given LCIA method. More emphasis has to be applied on this phase by assigning the proper qualitative or quantitative estimate for each inventory. One major step to reduce uncertainty is to have a characterization of data inventory. For that, selective indicators were chosen for the inputs. These indicators could be classified into two categories; one has to do with the appropriateness of the data such as flow rate, solids content, and spatial conditions. The other category has to do with the reliability of the data such as the date and its amount (Maurice et al., 2000). The inventory data was characterized with the main affecting factors including statistics of data (e.g. mean, typical, range, etc.), flow rate, solids content, retention time, technologies or treatments used, and age, source, and methods of collection. If some variation in data came from the influence of some factor(s), a correlation was investigated to assign the suitable data for the case study as indicated in the file “Supplementary Materials.doc”.

### 2.5 LCIA method selection

One of the most important steps in the LCA study is to choose the suitable LCIA method. The impact assessment method is a connection between the life cycle inventory with the impacts on humans and the environment. A way of making this connection is to classify the LCI results into impact categories depending on the effects they made to the human health and the environment.

LCIA methods illustrate the impact through two control stages: midpoint and endpoint. Information from LCI are consolidated at the midpoint stage and are reported as stressors in quantitative measures such as total amount of kg CO_2_ equivalent or kg N equivalent from all of LCI. The damages on human health, ecosystem, and the natural resources are described at the endpoint stage, and are reported in quantitative measures such as disability adjusted life years (DALYs, i.e. the number of years life lost and the number of years lived disabled), loss of species over a certain area during a certain time, and expenses needed by future generations to extract resources depleted by us. Both midpoint and endpoint stages are considered in the reported research of this paper.

Many LCIA methods are available for LCA study such as Eco-indicator 99 (PRe, 2001) and IMPACT 2002 (Jolliet et al., 2003). Lazarova et al. (2012) reported the use of Eco-indicator 99, CML2000, and Ecological Scarcity for LCA analyses of water reuse processes. It was recommended that LCIA method should be able to cover most of the impact categories concerned, and produce and report LCA in a single score for easy comparison among evaluated options (Hung and Ma, 2009; Mimoso et al., 2015; Pizzol et al., 2011). Choosing such method will help discover elements that have high overall contribution to the impacts and ease performing more data collection analysis. After running a preliminary analysis and calculating the percentage contribution to the single score for every inventory, an LCA practitioner may perform more data collection for those inventories with high contribution in order to reduce uncertainty and gain more confidence in data collected and outcome resulted.

ReCiPe was an upgraded method from the Eco-indicator 99 (Goedkoop et al., 2013). The ReCiPe method reports the impacts on the environment or health in 17 categories. These 17 impact categories include the most relevant to the impacts resulted from wastewater treatment processes such as human toxicity, particulate matter or photochemical oxidant formation, fresh water eutrophication, marine eutrophication, and terrestrial acidification. ReCiPe method is able to produce a weighted single score for reporting the overall LCA impact. ReCiPe was chosen as the LCIA method for this research.

Hung and Ma (2009) showed that the Eco-indicator 99 has the lowest uncertainty while EDIP method had the highest. Some studies showed high uncertainties from LCIA method especially in the toxicity related impact categories making it difficult to drive clear conclusions from LCA without careful selection of LCIA (Pizzol et al., 2011). To consider the uncertainty in LCIA methodology, an LCA practitioner had to test results with more methods as possible; and the following steps can be used:

1- If the concern was one of the midpoint impact categories then comparison should be made between these categories. Due to difference between categories used by different methods, LCA may focus on only similar categories with similar units. LCA practitioner should pay attention only to categories that have high contribution to the total impact.
2- If the concern was the overall impact on environment then comparison should be made between damage categories and single score at the endpoint level. If results show some differences, these differences should be explained to decision makers with recommendations. Although, calculating single scores will need a weighting step which adds another type of uncertainty, it is necessary for comparison in this level. The weighting used will depend on selected cultural perspective. The model uncertainty due to cultural perspective will not be discussed in this paper.

### 2.6 Identifying major parameters in LCI

Literature review and expert opinions are essential in knowing what the contributors to be in the inventory are. The inventory for each scenario was built. Then, the results of models were used to identify major contributors using a single score indicator. An inventory with a single score contribution higher than 1% of the total impact in a model was considered major contributor, while those with a contribution less than 1% were considered minors. Major parameters for this research included natural gas and electricity consumed, energy produced from biogas, and chemicals (e.g. polymer) for dewatering. These parameters were selected for more data analysis to ensure accuracy as can be found in the file “Supplementary Materials.doc”.

When LCI for a major parameter is incomplete, the following methods are used:

1) In some cases, the principle of similarity between two processes can be used. For example, due to the lack of information of the steam electric heat rate in the process of sludge incineration, the heat rate from coal-fired power plant can be used due to similarity in principle of the two processes.
2) Applying mass balance to fill data gap of missing values (Maurice et al., 2000; Huijbregts et al., 2001). For example, mass conservation could be used to estimate the amount of bottom ash resulting from sludge incineration depending on the percentage of fixed solids.
3) Using ingredient data to calculate. For example, NO_x_ emissions from wastewater treatment processes can be determined from fuel consumption and are calculated by using the IPCC methods (Steinacher, 2011).
4) Using database comes with the LCA software (e.g. selected data in the Ecoinvent database that are available in SimaPro software were used to fill in data gap of air and water emissions from wastewater sludge treatment process) (Doka, 2006).

If a parameter is relevant to the studied process, but the researchers don't consider the parameter as equally significant as those major parameters, the parameters are put in the category of minor parameters and are handled in the following methods.

1) Neglecting from the LCI if not often found. E.g. asbestos are not normally present in wastewater sludge, hence asbestos are not included in incineration emissions (Nueman et al., 2012).
2) Including regulated parameters. E.g., it is recognized that wastewater treatment LCA studies did not follow consistent criteria what to include or what to exclude in terms of air emissions from incineration (Lundin et al., 2004). Hong et al. (2009) included CO_2_, N_2_O, dioxin, and emitted metals of Cr, Pb, Cd, Hg, and Zn from FBI process. The LCA study by Lederer and Rechberger (2010) on FBI included emitted metals of As, Cd, Cr, Cu, Hg, Ni, Pb, and Zn. Hospido et al. (2005) included CO_2_, CO, NO_2_, total particular matters (PM), dioxin/furan from incineration, but didn't consider any of metal emissions. In some cases, the inconsistency comes from the difference in the impacts that were assessed. Murray et al. (2008) evaluated limited number of impacts and included only CO_2_, CO, NO_x_, CH_4_, and SO_2_ in their study of the FBI. The recommended approach is to include all parameters whose emissions are regulated; regardless of how much are these parameters may contribute to the impact. Applicable local, regional, or international regulations are to be considered. The US *Clean Air Act* Section 129 requires EPA to set emission limits of nine pollutants from *Sewage Sludge Incineration* facilities: cadmium, carbon monoxide, dioxins/furans, hydrogen chloride, lead, mercury, NOx, particulate matter, and SO_2_. These nine pollutants are all included in LCI of MHI and FBI. Additional parameters may be included if LCI data are readily available from wastewater plant or literature.

### 2.7 Estimating uncertainty of LCI data

The uncertainty analyses by Monte Carlo simulation incorporate the variation in data values of the considered parameters. Because available data can be in any form in terms of how many data points and distributions, suitable mathematical model to describe the distribution of each parameter's values becomes necessary. Common distributions for parameters associated with wastewater treatment processes are normal or lognormal distribution (Oliveira et al., 2012). In LCA's Ecoinvent database, all data follow lognormal distribution. For normal distribution, two times of standard deviations cover 95% confidence interval and the arithmetic mean represents the best guess value. For lognormal distribution, the geometric mean divided and multiplied by the square of geometric standard deviation cover 95% confidence interval and the geometric mean represents the best guess value.

When adequate number of data points is available, a goodness-of-fit test was conducted to verify the mathematical distribution and determine the best guess value and the standard deviation. Vose (1996) suggested that, when 30 or more data points are available, the goodness-of-fit test should be conducted. For this study, routinely monitored data at BPWWTP such as treatment flow rate, amount of incinerated sludge, consumed natural gas, electricity, and chemicals for dewatering all provided adequate number of data points and were tested. The analysis using Anderson-Darling (A-D) statistics method revealed these parameters generally followed lognormal distribution.

On the other hand, when the number of available data is inadequate for running goodness-of-fit test, alternative approach was considered. Kennedy et al. (1996, 1997) suggested taking (+/-50%) of available data's range as upper and lower limits. Maurice et al. (2000) suggested using large intervals when small number of data is available. When available data are reported as a range, or a range with a typical value, instead of an average or distribution, it is assumed that the data follow a lognormal distribution, and the best guess value is defined as the geometric mean that is calculated from the minimum and maximum values of the reported range.

When available data are reported as only a typical value, data's uncertainty can be estimated using a data quality approach, which use data quality indicators to develop additional data points for each parameter. These indicators are semi-quantitative numbers that specify the data quality in relation to the way it was used in the study (Weidema and Wesnaes, 1996). The following equation is used to calculate the square geometric standard deviation. The seven indicators in the equation define the uncertainty factor of a specific aspect. These seven indicators arranged in as described by PRe (2010), Weidema and Wesnaes (1996), Frischknecht et al., 2007.

$$SD_{g95}=\sigma^{2}=\mathrm{ex}p^{\sqrt{{\left(\ln\left(U1\right)\right)}^{2}+{\left(\ln\left(U2\right)\right)}^{2}+{\left(\ln\left(U3\right)\right)}^{2}+{\left(\ln\left(U4\right)\right)}^{2}+{\left(\ln\left(U5\right)\right)}^{2}+{\left(\ln\left(U6\right)\right)}^{2}+{\left(\ln\left(U7\right)\right)}^{2}+{\left(\ln\left(\mathrm{Ub}\right)\right)}^{2}}}$$

Where these are uncertainty factors of: U_1_: reliability, U_2_: completeness, U_3_: temporal correlation, U_4_: geographic correlation, U_5_: other technological correlation, U_6_: sample size, U_7_: added human error factor, and U_b_: basic uncertainty factor. Of these data quality indicators, five were suggested by Weidema and Wesnaes (1996). These indicators are reliability, completeness, temporal, geographical, and further technological correlation. Ecoinvent database adopted these indicators and added sample size as an additional indicator. This study added another indicator to cover uncertainty from human errors. These seven indicators arranged in a Pedigree Matrix as described by (PRe, 2010). The basic uncertainty factor is determined by experts for observed range of each parameter. The default values of these indicators are shown in the Pedigree Matrix described in PRe (2010). The LCI data of the three studied processes are shown in the “Supplemental Materials.doc” file.

Another type of uncertainty analysis is to test sensitivity of results to qualitative selection of probability distributions when enough data are not available. For instance, uniform, triangular and normal distributions could be used as part of a sensitivity analysis, instead of utilizing lognormal distributions only (normative, subjective). However, due to size limitation in this paper, such sensitivity analysis will not be performed.

### 2.8 Monte Carlo simulation for uncertainty estimation

Because values of LCA parameters are distributed in its respective range, only using the deterministic means for results evaluation and comparison is inadequate. Comparison will have to be conducted in all of possible combinations through Monte Carlo simulations.

When comparing different scenarios, it is easy to make the selection when results show a significant difference, but when the upper and lower limits of the scenarios’ results are overlapped this may reduce the confidence in conclusions made from results. For this reason, SimaPro does not show the overlapped distributions as this can easily lead to a wrong interpretation. Instead, SimaPro uses A-B approach. This approach calculates the difference between scenario A and scenario B in every iteration, and then shows in how many times scenario A scored lower than scenario B. when deal with more than two scenarios, SimaPro suggested using pairing simulation between the scenarios then extrapolate the preferred scenario from these comparisons.

This approach is time-consuming especially when there are many scenarios. As an example, to compare six scenarios, 15 comparisons are needed. Alyaseri (2014) suggested reducing the number of Monte Carlo comparisons through: 1) running Monte Carlo simulation for each scenario separately, 2) combining the probability distributions of outcomes (in the impact and damage categories and for the single score) for all scenarios together to see the overlapping, and 3) if two or more of the scenarios are overlapped in most of the life cycle stages (impact assessment, damage assessment and single score), then comparative Monte Carlo simulation can be performed for them.

Monte Carlo simulation offers the capability and simplicity to produce probabilistic results (Heijungs and Huijbregts, 2004; Huijbregts et al., 2001), hence it was chosen for quantify uncertainty for this research. The method was referred to as a promising technique due to the ability to simulate various parameters distributions such as uniform, normal, or lognormal in the model (Huijbregts, 1998), and the ability to perform all types of operations such as multiplications or matrix calculations (Maurice et al., 2000). LCA software SimaPro 8.0 has a built-in Monte Carlo simulation capability and was used for all analysis. Because three wastewater sludge processes (MHI, FBI, and AD) were studied, pairing simulation of MHI vs. FBI; MHI vs. AD; and FBI vs. AD were conducted. Simulation results were analyzed further for comparative evaluation among the three processes.

To test uncertainty from other LCIA methods, MC simulation between alternatives was performed using two other endpoint LCIA methods; Eco-indicatior 99, and Impact +2002. Owsianiak et al., 2014, show that differences between LCIA methods may comes from differences in; 1) the underlying characterization model, 2) substance coverage, 3) relative ranking of the reference substance, 4) spatial scales and reference years for normalization references, 5) approaches to weighting the differences between methods, and 6) due to combining similar compounds into a group with single characterization factor. Stavropoulos et al. (2016) show that the single score resulted from each LCIA method cannot be directly compared with the other due to differences in characterization, normalization, and weighting factors used in each method. Hence, when it comes to evaluate outcomes from different methods, impact or damage categories will be used for comparison instead of comparison based on single scores.

**Fig. 4:**
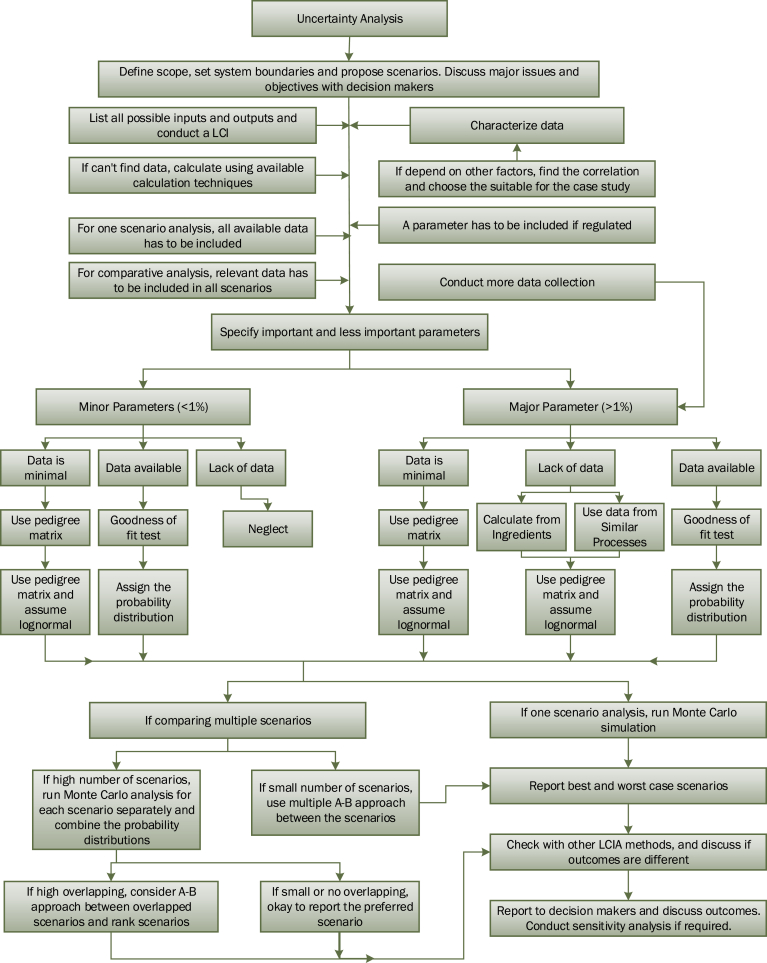
Organization chart of the uncertainty analysis procedure used by LCA of wastewater sludge treatment processes in WWTP.

The procedure of uncertainty analysis used in this study can be summarized in Fig. 4.

## 3 Results and discussion

### 3.1 Analysis of MHI process

LCI parameters are defined as distributed values: the values of each parameter vary in its respective range due to uncertainty. Monte Carlo simulations were conducted on each of three wastewater sludge treatment processes. For each process, the Monte Carlo analyses went through over 5,000 simulations resulted from combination of assorted values of included LCI parameters. The deterministic means were calculated results based on the average value of each parameter. The probabilistic means are based on the Monte Carlo simulations. The analysis results on MHI process are shown in Table 1.

**Table 1:** Monte Carlo Simulation Results for **MHI** Process: Characterization, Damage Assessment, and Single Score (One kg of Dry Mass of Sludge. ReCiPe Method, 95% Confidence Interval).

Impact category	Unit	Deterministic mean	Probabilistic Mean	Median	SD∗	CV∗∗	2.5%	97.5%
Climate change (human health)	DALY E-07	4.74	4.77	3.43	4.40	92.2	1.24	15.7
Ozone depletion	DALY E-11	1.88	1.90	1.35	1.65	86.9	0.61	6.02
Human toxicity	DALY E-07	2.16	2.16	1.85	1.20	55.5	0.86	5.16
Photochemical oxidant formation	DALYE-10	1.15	1.16	1.09	0.40	34.0	0.66	2.13
Particulate matter formation	DALYE-07	3.62	3.67	3.21	1.73	47.1	1.90	8.17
Ionizing radiation	DALYE-09	1.41	1.41	0.92	1.49	105	0.27	5.18
Climate change (ecosystems)	species.yrE-09	2.69	2.70	1.94	2.49	92.2	0.70	8.89
Terrestrial acidification	species.yrE-11	3.13	3.18	2.72	1.69	53.2	1.52	7.48
Freshwater eutrophication	species.yrE-12	8.81	8.72	5.84	11.7	134	2.14	31.0
Terrestrial ecotoxicity	species.yrE-11	3.06	3.00	2.69	1.40	46.8	1.24	6.60
Freshwater ecotoxicity	species.yrE-12	1.53	1.56	1.25	1.13	72.1	0.49	4.36
Marine ecotoxicity	species.yrE-15	3.68	3.68	3.07	2.23	60.5	1.53	9.33
Agricultural land occupation	species.yrE-11	4.20	4.22	2.79	4.35	103	0.87	15.2
Urban land occupation	species.yrE-11	7.43	7.44	6.07	4.42	59.4	3.99	18.7
Natural land transformation	species.yrE-12	5.06	5.47	-3.58	30.6	560	-22.6	80.8
Metal depletion	$ E-04	5.71	5.93	5.50	1.64	27.6	4.15	10.4
Fossil depletion	$	2.80	2.86	2.27	2.15	75.1	0.82	8.17
Ecosystems	species.yr E-09	2.88	2.87	2.17	2.55	89.0	0.74	9.50
Human health	DALY E-06	1.05	1.05	0.88	0.66	62.6	0.44	2.71
Resources	$	2.80	2.80	2.30	1.90	68.1	0.75	7.68
Single score	PtE-02	5.79	5.80	4.80	3.70	63.4	2.10	15.1

∗ SD: arithmetic standard deviation.

∗∗ CV: coefficient of variance. All columns except CV follow the specified unit. CV unit is in %.

The Table shows detailed statistical information for every impact or damage category which makes it easier to assess the best and worst cases in the environmental performance of the plant. For example, under the *Climate change (human health)* category, the 95% confidence interval range is between 1.24E-07 DALY and 15.7E-07 DALY, a large change of 12.7 times when the higher limit is compared to the lower limit. In a 95% confidence interval, the table shows damages between 0.44E-06 to 2.71E-06 DALY on human health and between 0.74E-09 to 9.50E-09 species.yr on ecosystem may occur due to MHI treatment for every one kg of sludge treated in the plant. With uncertainty analysis, decision makers are not limited to the deterministic results only but able to see worst and best scenarios related to the studied process.

The results revealed that the impact resulted from fossil fuel depletion has a probabilistic mean of $2.86 (i.e. extra cost comparing to today's effort, when the future generation extracts fossil fuels). The Table shows that the impact from fossil fuel is significantly higher than the impact from metals depletion ($2.86 vs. $5.93E-04).

The uncertainty analysis may also help in estimating annual damages. When consider annual tons of dry sludge incinerated (use year 2011 as reference), probabilistic mean damage on human health, ecosystem quality, and resources become 40.2 DALY (ranged from 16.7 to 103.8 DALY), 0.11 species-yr (ranged from 0.03 to 0.36 species-yr), and $107.3×10^6^ (ranged from $28.8×10^6^ to $294.1×10^6^), respectively.

### 3.2 Analysis of FBI and AD processes

Monte Carlo analysis results of FBI process are shown in Table 2. Positive values indicate anticipated impact (i.e. undesirable effect) on the environment or human health, while negative values indicate negative impact (i.e. beneficial effect mainly due to utilizing heat from incinerator to generate electricity).

**Table 2:** Monte Carlo Simulation Results for **FBI** Process: Characterization, Damage Assessment, and Single Score (One kg of Dry Mass of Sludge. ReCiPe Method, 95% Confidence Interval).

Impact category	Unit	Deterministic mean	Probabilistic mean	Median	SD∗	CV∗∗	2.5%	97.5%
Climate change (human health)	DALY E-07	2.38	2.37	1.85	5.02	212	-5.50	13.9
Ozone depletion	DALY E-11	-7.74	-7.78	-6.16	11.3	-146	-34.60	10.0
Human toxicity	DALY E-07	-2.26	-3.52	-2.75	5.37	-153	-16.2	4.96
Photochemical oxidant formation	DALY E-10	-0.23	-0.24	-0.16	1.04	-439	-2.53	1.57
Particulate matter formation	DALY E-07	0.48	0.47	0.45	2.18	462	-3.67	4.94
Ionizing radiation	DALY E-09	-0.28	-0.78	-0.59	1.65	-212	-4.54	1.92
Climate change (ecosystems)	species.yr E-09	1.35	1.34	1.05	2.84	212	-3.12	7.89
Terrestrial acidification	species.yr E-11	0.77	0.77	0.60	1.68	219	-1.88	4.62
Freshwater eutrophication	species.yr E-12	2.48	-30.9	-24.5	50.8	-164	-146	48.3
Terrestrial ecotoxicity	species.yr E-11	-0.29	-0.30	-0.14	1.16	-386	-3.01	1.55
Freshwater ecotoxicity	species.yr E-12	0.05	-0.87	-0.67	1.45	-167	-4.29	1.41
Marine ecotoxicity	species.yr E-15	0.09	-2.61	-1.98	4.56	-175	-13.4	4.54
Agricultural land occupation	species.yr E-11	-2.87	-2.88	-2.22	4.63	-161	-13.8	4.47
Urban land occupation	species.yr E-11	-0.18	-0.18	0.58	5.33	-3003	-12.8	8.32
Natural land transformation	species.yr E-12	9.04	9.49	8.53	14.3	151	-16.1	40.3
Metal depletion	$ E-04	4.75	4.96	6.12	10.0	202	18.0	21.5
Fossil depletion	$	1.05	1.05	0.75	1.77	169	-1.35	5.37
Ecosystems	species.yr E-09	1.34	1.29	1.03	2.94	228	-3.51	7.99
Human Health	DALY E-06	0.06	-0.07	-0.007	1.13	-1644	-2.51	1.99
Resources	$	1.05	1.05	0.75	1.77	169	-1.35	5.37
Single score	Pt E-02	1.19	0.80	0.78	4.90	610	-8.64	10.7

∗ Standard Deviation

∗∗ Coefficient of Variation. All columns except CV follow the specified unit. CV unit is in %.

As shown in the forth column in Table 2 (the probabilistic means), although FBI process resulted in impact on the environment or human health in a few sub-categories such as climate change human health (2.37E-07 DALY) and particulate matter formation (4.72E-08 DALY), many sub-categories such as ozone depletion (-7.78E-011 DALY), human toxicity (-3.52E-07 DALY), and ionizing radiation (-7.80E-10DALY) are in negative values, indicating beneficial effect on human health. When the probabilistic means in Table 2 (FBI process) are compared to those in Table 1 (MHI process), all values of FBI process (e.g. climate change human health 2.37E-07 DALY) are lower than that of MHI process (e.g. climate change human health 4.77E-07 DALY), indicating the FBI process is a more environmental favorable process.

Monte Carlo analysis further revealed the impact of uncertainty of LCI input data on the LCA output. For example, the damages on ecosystem quality range from the best scenario of saving 3.51E-09 species-yr to the worst scenario of damaging 7.99E-09 species-yr. The impacts on human health range from the best scenario of avoiding 2.51E-06 DALY to the worst scenario of causing 1.99E-06 DALY. Because selecting a more desirable and sustainable treatment process are influenced by findings from LCA analysis, the wide range of LCA results revealed the essential need and significance of adequately addressing the impact of uncertainty on process evaluation.

The Monte Carlo analysis results of AD process are shown in Table 3. Similar to the FBI process, the AD process resulted in impact on the environment or human health in a few sub-categories such as human toxicity (probabilistic mean of 1.38E-8 DALY) and urban land occupation (probabilistic mean of 1.34E-11 DALY). However, the probabilistic means in many sub-categories such as ozone depletion (-4.87E-11 DALY), climate change ecosystems (-1.24E-09species-yr), and ionizing radiation (-1.29E-08 DALY) are in negative values, indicating beneficial impact on the environment. When the probabilistic means in Table 3 (AD process) are compared to those in Table 2 (FBI process), AD process (e.g. climate change human health -2.20E-07 DALY) are lower than that of FBI process (e.g. climate change human health +2.37E-07 DALY), indicating the AD process is an even more favorable process in terms of the impact on human health.

**Table 3:** Monte Carlo Simulation Results for **AD** Process: Characterization, Damage Assessment, and Single Score (One kg of Dry Mass of Sludge. ReCiPe Method, 95% Confidence Interval).

Impact category	Unit	Deterministic mean	Probabilistic mean	Median	SD∗	CV∗∗	2.5%	97.5%
Climate change (human health)	DALY E-07	-2.22	-2.20	-1.29	3.90	-178	-11.9	2.46
Ozone depletion	DALY E-11	-4.91	-4.87	-4.08	3.27	-67	-.3.4	-1.07
Human toxicity	DALY E-07	0.05	0.14	0.18	0.21	155	-0.38	0.43
Photochemical oxidant formation	DALY E-10	0.26	0.27	0.29	0.19	73	-0.19	0.58
Particulate matter formation	DALY E-07	3.20	3.20	3.12	1.01	32	1.41	5.43
Ionizing radiation	DALY E-09	-0.48	-1.29	-0.84	1.66	-128	-5.15	-0.21
Climate change (ecosystems)	species.yr E-09	-1.25	-1.24	-0.72	2.21	-178	-6.75	1.40
Terrestrial acidification	species.yr E-11	4.90	4.91	4.72	1.57	32	2.39	8.51
Freshwater eutrophication	species.yr E-12	-0.02	11.0	10.4	3.83	35	4.91	19.8
Terrestrial ecotoxicity	species.yr E-11	-0.09	-0.09	-0.07	0.10	-109	-0.33	-0.002
Freshwater ecotoxicity	species.yr E-12	0.008	-0.10	-0.07	0.12	-124	-0.41	0.04
Marine ecotoxicity	species.yr E-15	-0.02	-0.35	-0.26	0.39	-111	-1.35	0.11
Agricultural land occupation	species.yr E-11	-0.22	-0.19	-0.08	0.46	-242	-1.34	0.38
Urban land occupation	species.yr E-11	1.30	1.34	1.89	2.68	201	-5.55	4.88
Natural land transformation	species.yr E-12	-110	-110	-103	43.7	-40	-214	-47.3
Metal depletion	$ E-04	-6.3	-6.3	-5.0	5.40	-86	-20.3	-0.02
Fossil depletion	$	-0.06	-0.05	0.07	0.53	-1022	-1.43	0.60
Ecosystems	species.yr E-09	-1.30	-1.28	-0.76	2.25	-176	-6.94	1.43
Human Health	DALY E-06	-0.10	0.11	0.20	0.45	401	-1.03	0.70
Resources	$	-0.06	-0.05	0.07	0.53	-1010	-1.43	0.60
Single score	Pt E-02	0.19	0.23	0.62	1.85	817	-4.46	2.62

∗ Standard Deviation.

∗∗ Coefficient of Variation. All columns except CV follow the specified unit. CV unit is in %.

Monte Carlo analysis revealed the impact of uncertainty of LCI input data on the LCA output. For example, the damages on ecosystem quality range from -6.94E-09 species-yr to +1.43E-09 species-yr. The damage on human health range from -1.03E-06 DALY to +6.98E-06 DALY. LCA results from AD process analysis revealed further the significance of uncertainty impact on process evaluation.

### 3.3 Comparative analysis of MHI, FBI and AD processes

The three studied processes were compared in pairs: FBI vs. MHI, FBI vs. AD, and MHI vs. AD. Comparison between the FBI and the MHI show that in all impact categories, except the impact on natural land transformation, there is high probability to have lower impacts from FBI. The FBI has more preference on the MHI. There are 82.4%, 70.9%, and 84.2% probabilities to have lower damages on resources’ depletion, ecosystem quality, and human health, respectively when using the FBI to treat sludge.

Comparison between AD and MHI shows that the former scenario has better performance in all impact categories except in acidification and eutrophication and this is mainly due to higher percentage of solids (digestate) taken to the landfill. The AD showed better performance on the three damage categories (99.9%, 98.3%, and 97.0% probability to have lower damages on resources’ depletion, ecosystem quality, and human health, respectively when using AD).

**Fig. 5:**
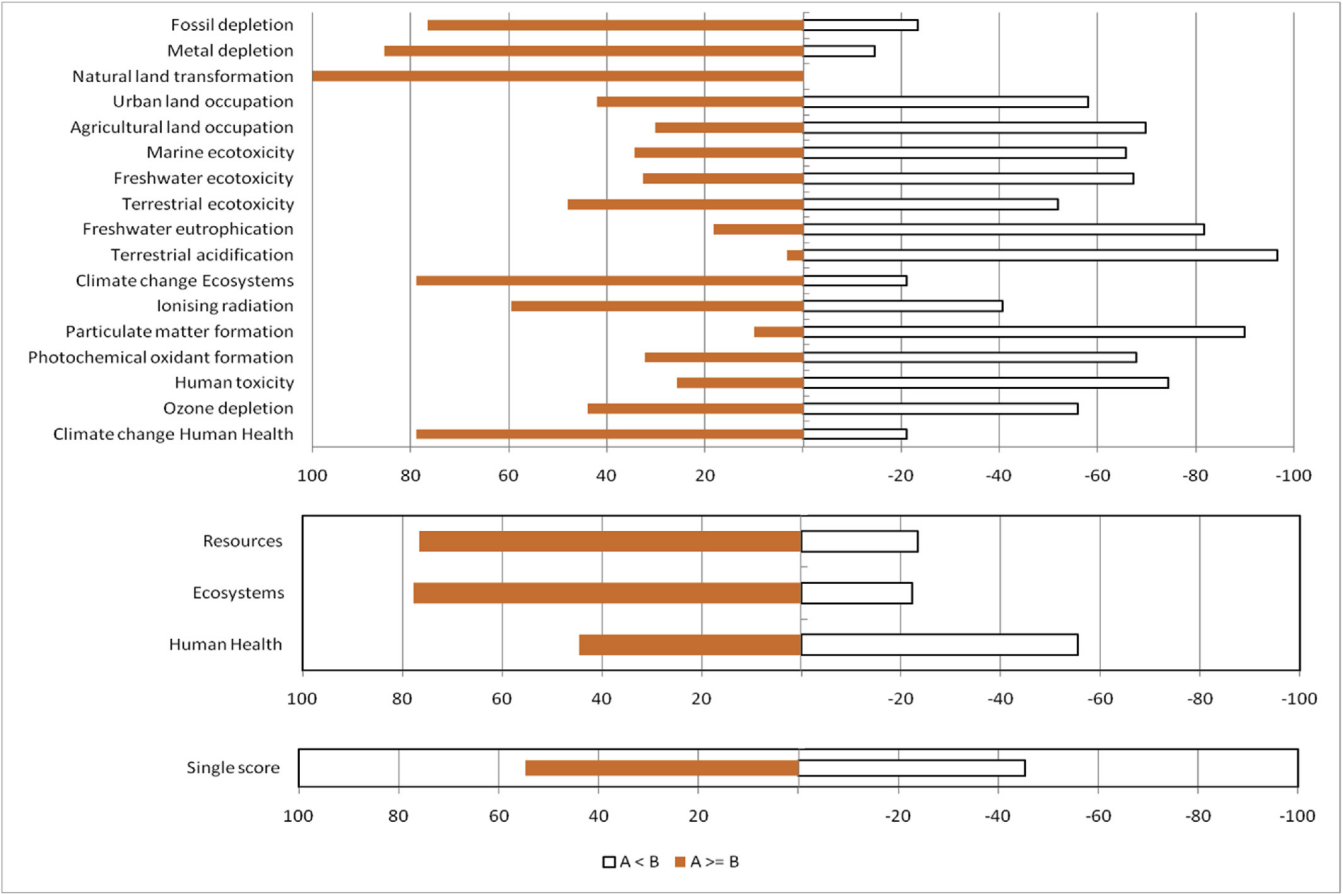
Monte Carlo Comparison between FBI (A in the graph) and AD (B in the graph) processes. A positive value (red bar on the left) indicates the percentage among all analyzed scenarios that FBI results in higher impact on the environment than AD (i.e. FBI is a less desirable process). A negative value (open bar on the right) indicates the percentage among of analyzed scenarios that FBI results in lower impact on the environment than AD (i.e. FBI is a more desirable process). The left and right percentages are added to be 100%.

Results shown in Fig. 5 reveal that, FBI minus AD processes show positive values in several sub-categories such as +78.9% in climate change human health, +78.0% in climate change ecosystems, and +100% in natural land transformation, meaning FBI would result in higher environmental damages than AD process in these categories. Therefore, if the priority concerns are about the impact of the climate change and natural land, FBI is a less desirable process when comparing to AD process. However, the use of FBI process results in less damage on the environment or human health in many of other sub-categories when the positive values shown in Fig. 5 are less than 50%.

For the three categories of resources, ecosystems, and human health, the FBI minus AD percentages are +76.3%, +76.6%, and +44.6%, respectively. Overall, FBI minus AD is +54.7% in terms of single score. Therefore, there is more possibility (more than half of time) that the FBI process creates higher damages than the AD process.

Results of Monte Carlo simulation to compare FBI and AD processes are shown in Table 4. In addition to the differences in terms of percentages when FBI process (A in Table 4) creates higher damages on the environment or human health than AD process (B in Table 4), the differences in terms of probabilistic means and at both ends of 95% confidence intervals (i.e. 2.5%–97.5%) are also shown in Table 4. Take the ecosystem quality as an example, analysis revealed that there is 77.8% possibility that FBI process creates higher damage than AD process. However, at 2.5% limit, FBI process minus AD process shows -3.34E-09, indicating FBI process could be taken as a process of lower damage on ecosystems when comparing to AD process, when the confidence level is decreased to lower end. Therefore, decision maker for selecting an environmentally sound process needs to be aware how selection criteria such as confidence interval and uncertainty associated with LCI data.

**Table 4:** Monte Carlo Analysis Probabilistic Comparison between **FBI** (A in the table) and **AD** (B in the table) processes. The percentage indicated the probability among all combination scenarios when **FBI** process has higher impact than **AD** process in the categories. The higher than 50% probabilistic percentages are associated with positive probabilistic means, indicating FBI process causes higher impact on the environmental or human health (i.e. **FBI** is a less desirable process).

Impact category	A ≥ B %	Unit	Probabilistic Mean	Median	SD∗	CV∗∗	2.50%	97.50%
Climate change (human health)	78.9	DALY E-07	4.56	3.66	6.36	140	-5.24	19.7
Ozone depletion	44.0	DALY E-11	-2.96	-1.58	11.9	-403	-30.9	16.4
Human toxicity	25.7	DALY E-07	-3.64	-2.87	5.44	-149	-16.6	4.75
Photochemical oxidant formation	32.2	DALY E-10	-0.50	-0.42	1.07	-214	-2.85	1.40
Particulate matter formation	10.1	DALY E-07	-2.72	-2.72	2.41	-89	-7.51	2.26
Ionizing radiation	59.5	DALY E-09	0.52	0.38	2.68	519	-3.87	6.27
Climate change (ecosystems)	78.9	species.yr E-09	2.58	2.07	3.60	140	-2.97	11.1
Terrestrial acidification	3.4	species.yr E-11	-4.13	-4.13	2.28	-55	-8.56	0.51
Freshwater eutrophication	18.3	species.yr E-12	-42.0	-35.7	51.5	-123	-160	34.0
Terrestrial ecotoxicity	48.1	species.yr E-11	-0.21	-0.05	1.18	-567	-3.02	1.63
Freshwater ecotoxicity	32.7	species.yr E-12	-0.77	-0.56	1.47	-193	-4.26	1.51
Marine ecotoxicity	34.4	species.yr E-15	-2.25	-1.62	4.64	-206	-13.3	4.91
Agricultural land occupation	30.2	species.yr E-11	-2.70	-2.08	4.71	-174	-14.0	4.56
Urban land occupation	42.0	species.yr E-11	-1.52	-1.05	6.03	-398	-15.0	9.19
Natural land transformation	100.0	species.yr E-12	119	113	42.9	36	54.9	218
Metal depletion	85.4	$ E-04	11.2	11.8	11.8	106	-13.8	32.4
Fossil depletion	76.6	$	1.10	0.79	1.82	166	-1.54	5.65
Ecosystems	77.78	species.yrE-09	2.57	2.09	3.71	145	-3.34	11.3
Human Health	44.6	DALYE-06	-0.18	-0.14	1.24	-688	-2.75	2.21
Resources	76.63	$	1.10	0.79	1.82	166	-1.54	5.65
Single score	54.69	Pt E-02	0.58	0.49	5.27	904	-0.10	11.7

∗ Standard Deviation.

∗∗ Coefficient of Variation. All columns except CV follow the specified unit. CV unit is in %.

### 3.4 Comparative analysis of different LCIA methods

Table 5 show the Monte Carlo comparison between the two alternatives for three endpoint LCIA methods; ReCipe 2008, Eco-indicatior 99, and IMPACT +2002. On the single score level, the two later methods favor FBI over AD. On the damage assessment level, in contradict with the ReCipe 2008 and IMPACT 2002 + methods, Ecoindicator 99 shows favorability to FBI over AD in the ecosystem quality category. The results from other damage categories are consistent from the two methods. This difference in ecosystem quality category comes from difference in characterization factors employed in every method. In general, the endpoint characterization factors derived by ReCipe for ecosystem quality indicators are systematically higher compared to eco-indicator 99 (Stavropoulos et al., 2016).

**Table 5:** Uncertainty results of comparison between the scenario of treatment using FBI (A) and the scenario of treatment using AD (B) on the impact assessment in characterization, damage assessment, and single score stages using three endpoint LCIA methods.

ReCipe H endpoint		Eco-indicator 99		Impact 2002 + v2.1	
Impact category	A ≥ B	Impact category	A ≥ B	Impact category	A ≥ B
				Global warming	76.36
Climate change Ecosystems	78.9	Climate change	78.9		
Climate change Human Health	78.9				
Fossil depletion	76.6	Fossil fuels	79.2	Non-renewable energy	75.67
Freshwater ecotoxicity	32.7	Ecotoxicity	29.9	Aquatic ecotoxicity	0
Marine ecotoxicity	34.4				
Terrestrial ecotoxicity	48.1			Terrestrial ecotoxicity	100
Freshwater eutrophication	18.3			Aquatic eutrophication	69.49
Human toxicity	25.7	Carcinogens	38.2	Carcinogens	23.64
Particulate matter formation	10.1	Resp. inorganics	14.7	Respiratory inorganics	12.34
Photochemical oxidant formation	32.2	Resp. organics	59.4	Respiratory organics	44.23
				Non-carcinogens	24.37
Ionising radiation	59.5	Radiation	58.7	Ionizing radiation	59.27
Ozone depletion	44.0	Ozone layer	39.5	Ozone layer depletion	47.36
Metal depletion	85.4	Minerals	85.9	Mineral extraction	89.4
Terrestrial acidification	3.4	Acidification/Eutrophication	0.2	Aquatic acidification	8.01
				Terrestrial acid/nutri	0.26
Natural land transformation	100.0	Land use	70.0	Land occupation	67.99
Urban land occupation	42.0				
Agricultural land occupation	30.2				
				Climate change	76.36
Ecosystems	77.78	Ecosystem Quality	6.8	Ecosystem quality	98.06
Human Health	44.6	Human Health	28.0	Human health	18.25
Resources	76.63	Resources	79.8	Resources	75.71
Single score	54.69	Single score	36.5	Single score	45.85

The ReCipe Endpoint is more justified and recommended to use for the assessment of the damage to ecosystem quality and human health, as it provides more indicators, and combines the scientific rigor of CML2001 and the simple interpretation of the results in Eco-indicator 99 (Verbitsky and Pushkar, 2018; and Stavropoulos et al., 2016). Studies show that different impact assessment methods are expected to provide congregate results if one process was the main driver of environmental impact (Huijbregts et al., 2010) but with multiple processes in air, water, and soil under ecosystem quality category, the difference in outcome is expected. For acidification and eutrophication, the direct comparison is not possible because ReCiPe 2008 does not include impacts on terrestrial eutrophication, whereas the IMPACT 2002 + combines impacts on terrestrial acidification with impacts on terrestrial eutrophication and the Eco-indicator 99 combined acidification and eutrophication in one impact category. Emissions of ammonia and nitrogen oxides from AD are lot more than from FBI resulted in favorability of the later scenario in acidification category in all methods. Although nitrogen oxides and phosphate emission is more from AD scenario, there is difference between ReCiPe 2008 and IMPACT 2002+ in impact scores for aquatic eutrophication due to differences in the characterization models. The former includes substance fate and makes a distinction between limiting nutrients in aquatic bodies (P in freshwater and N in marine water), whereas midpoint coefficient factors in IMPACT 2002 + are those from the CML 2002 methodology where both substance fate and distinction between limiting nutrients are ignored (Owsianiak et al., 2014). Both ReCiPe 2008 and IMPACT 2002 + have terrestrial eco-toxicity as a separate impact category. Comparison shows discrepancy between the two methods. IMPACT 2002 + gives strong weight to impacts from metal emissions which led to favorability of AD scenario on the terrestrial eco-toxicity category.

## 4 Conclusions

This research evaluated the impact of uncertainties from LCI and LCIA method on LCA outcomes. One full-scale wastewater treatment plant using multiple hearth incineration (MHI), and two proposed alternative processes; fluid bed incineration (FBI) and anaerobic digestion (AD), were used for case study. It was found that FBI and AD processes produced lower environmental stressors than MHI process across all 17 subcategories. Monte Carlo simulations revealed that, when comparing to the anaerobic digestion process, the probabilities that fluid bed incineration process created higher negative impact on the environment are 76.3%, 76.6%, and 44.6% in the consolidated categories of resources, ecosystems, and human health, and are +54.7% in the final single score category. Therefore, uncertainty in LCI data results in the selection of an environmentally desirable process a probabilistic process instead of a deterministic process. This research proposed a process flowchart that LCA researchers can follow for systematic handling of uncertainties from LCI encountered by LCA studies. With respect to discovering the alternative with the lowest environmental impact, studied LCIA methods point out different conclusion. Hence, the choice of the LCIA methodology over the existing alternatives does matter especially if the concern was more for one of the impact categories related to ecosystem quality. Although this study was able to cover most elements in parameters and choices uncertainty, it didn't cover any of model uncertainty such as the uncertainty related to different cultural perspectives which leads to different methods of deriving emissions and characterization factors, and disagreement due to subjective judgment. Hence, further uncertainty analysis for this case study has to be performed to cover all possible scenarios before decision makers may make their decisions.

## Declarations

### Author contribution statement

Isam Alyaseri: Conceived and designed the experiments; Performed the experiments; Analyzed and interpreted the data; Wrote the paper.

Jianpeng Zhou: Conceived and designed the experiments; Analyzed and interpreted the data.

### Funding statement

This research did not receive any specific grant from funding agencies in the public, commercial, or not-for-profit sectors.

### Competing interest statement

The authors declare no conflict of interest.

### Additional information

No additional information is available for this paper.

## Appendix A Supplementary data

Supplementary Materials.doc Supplementary Materials.doc
